# Impact of Prolonged Absence of Organized Training on Body Composition, Neuromuscular Performance, and Aerobic Capacity: A Study in Youth Male Soccer Players Exposed to COVID-19 Lockdown

**DOI:** 10.3390/ijerph19031148

**Published:** 2022-01-20

**Authors:** Sümer Alvurdu, Cihan Baykal, Zeki Akyildiz, Ömer Şenel, Ana Filipa Silva, Daniele Conte, Filipe Manuel Clemente

**Affiliations:** 1 Faculty of Sport Sciences, Gazi University, 06500 Ankara, Turkey; sumeralvurdu@gazi.edu.tr (S.A.); cihanbaykal@gmail.com (C.B.); zekiakyldz@hotmail.com (Z.A.); osenel@gazi.edu.tr (Ö.Ş.); 2 Escola Superior Desporto e Lazer, Instituto Politécnico de Viana do Castelo, Rua Escola Industrial e Comercial de Nun’Álvares, 4900-347 Viana do Castelo, Portugal; anafilsilva@gmail.com; 3 Research Center in Sports Performance, Recreation, Innovation and Technology (SPRINT), 4900-347 Viana do Castelo, Portugal; 4 Institute of Sport Science and Innovations, Lithuanian Sports University, 44221 Kaunas, Lithuania; daniele.conte@lsu.lt; 5 Instituto de Telecomunicações, Delegação da Covilhã, 1049-001 Lisboa, Portugal

**Keywords:** football, athletic performance, detraining, stay at home orders, youth football

## Abstract

The aim of this study is to examine how physical performance has changed after 15 weeks (109 days) long-term absence of organized training in youth soccer players imposed by the stay at home orders. A total of sixty-eight young male soccer players from different age categories (U15, U16, U17 and U19) voluntarily participated in the prospective cohort study. Body fat percentage (BF%), counter-movement jump (CMJ), 30 m sprint, change-of-direction (COD) and yo-yo intermittent recovery test level-1 (YYIRTL-1) were evaluated twice (before and after the detraining period). Subsequently, 2 × 2 repeated measures ANOVA was used to investigate group and time differences in repeated measurements. A significance level of *p* < 0.05 was implemented. CV and SWC values were calculated to test the reliability of the tests performed at different times. Statistical analysis was performed using the IBM SPSS statistics software (v.25, IBM, New York, NY, USA). Significant increments in BF%, 30 m sprint, and COD (left and right), and also significant decrements in CMJ and YYIRTL-1, were found after the detraining period. A long-term detraining period due to the stay at home orders has a detrimental effect on body composition, neuromuscular performances, and aerobic capacity in youth soccer players.

## 1. Introduction

Detraining is a well-known and much studied physiological effect in the sports literature [1]. This term refers to the partial or complete loss of previous physiological adaptation to physical exertion caused by a period in which organized training is ceased or reduced [2]. In general, a few weeks of inactivity or lesser activity is sufficient for a decrease in physiological parameters unless specific training programs are carried out [3]. Detraining causes changes in body composition and a loss of activity in the neuromuscular and cardiovascular systems [4], and consequently a significant loss of performance in parameters such as strength, power, speed, flexibility, and endurance [5,6]. Moreover, training cessation seems to have a higher impact on the loss of body composition and fitness parameters compared to those who maintain some kind of individual training program [7]. 

Two types of detraining have been described in the literature: short-term (less than four weeks) and long-term (more than four weeks) [1,8]. In a general soccer season, there may be situations of cessation due to a break at the end of league competitions or due to an injury. This is the common type of short- and long-term detraining. However, it is not possible to compare this situation with the lack of training during the pandemic due to the coronavirus disease (COVID-19) lockdown [9,10] in which the training process may have been interrupted unexpectedly based on the stay at home orders.

Although there is an abundant literature on the measurement of young soccer athletes’ change in performance levels during the detraining process [6,11,12,13,14,15], studies on the effects of the COVID-19-pandemic-caused lockdown period on athletes’ physical performance levels are as yet very limited [16,17,18,19]. Studies specific to the COVID-19 pandemic period are mostly focused on strategies and recommendations that athletes can do at home and guidelines on how they should restart training [20,21,22,23,24,25,26].

The detraining period caused by the COVID-19 stay at home orders represents a great unknown about the physiological changes that occur in athletes. Although the lockdown periods differ from country to country, returning to training for athletes has followed a much longer detraining period than usual following the end of the regular season [9]. 

Following the outbreak of the COVID-19 pandemic, in parallel with the cancellation/postponement of tournaments and leagues in Europe and almost all over the world, the Turkish Super League was suspended from 19 March 2020 to 12 June 2020 and all grassroots training of the teams in professional leagues was suspended likewise.

At the end of this compulsory and long quarantine period, the intensity of the competition schedule that the athletes encounter upon returning to the game, not having an adequate pre-season preparation and being away from long-term training, can put the athletes under a much higher burden than normal and expose them to injury [7,15]. 

Therefore, in soccer, which has complex physiological requirements consisting of low, medium, and high intensity movements where aerobic and anaerobic metabolic demands are frequent [27], it is extremely important to measure and evaluate the data of physical performance parameters before returning to training. Although systematic information about the consequences of detraining in soccer players [12,14] is still needed to understand the effects of long-term detraining caused by unexpected conditions related to lockdown in which, besides the interruption of the training process, the duration has not been previously considered and the time is much longer than normal off-season periods that have been reported by the literature [7,15]. As an example, a systematic review with meta-analysis published recently (2021) in soccer revealed that from the 12 articles included, 11 (91.7%) were between 2 and 6 weeks (thus, between 21 and 42 days), and only one was above 6 weeks (in that case 12 weeks, 84 days) [7]. In the same review, from the 12 included studies, only 2 (approximately 17%) were conducted in players below 18 [7]. Thus, it is obvious that longer periods of detraining and studies conducted in youth soccer players are scarce and require more research. This creates additional importance for knowing the effects better in a youth who suffers a decrease in training stimulus and likely in regular physical activity levels in their regular life. Based on the above-mentioned reasons, the aim of this study is to examine how body composition, neuromuscular performance, and aerobic capacity of young soccer players (from U15 to U19) have changed after the prolonged absence of organized training due to the COVID-19 stay at home orders.

## 2. Materials and Methods

### 2.1. Experimental Approach to the Problem

**Figure 1:**
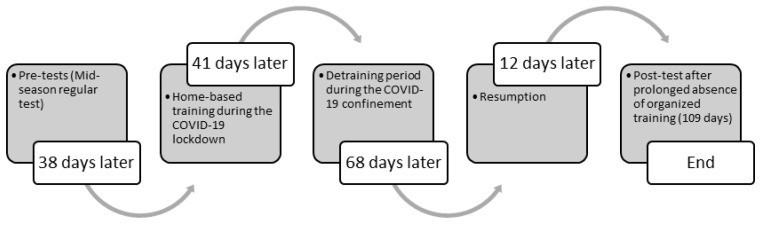
Schematic view of the study design.

A prospective observational study design was implemented for describing the variations of body composition and fitness parameters of youth soccer players from different age categories after 109 days of prolonged absence from organized training caused by the COVID-19 pandemic (Figure 1). 

The study design was chronological as follows:

- Mid-season measurements of the players were taken 38 days (26 training sessions) before the start of COVID-19 stay at home orders.
- During the COVID-19 lockdown (41 days), players performed a standardized 40 min home-based training program consisting of body-weight resistance exercises (i.e., dynamic movements and mobility exercises for warm-up and 3 sets of 10–15 repetitions of 6 conditioning workouts such as high knees, jumping jacks, lunges, squats, burpees, etc., and 6 core workouts such as planks, quadrupeds, hip bridges, crunches, push-ups, etc., with 30 s rest between exercises and 3 min recovery between sets). Players trained individually 3 times a week at home and were monitored online under the supervision of group coaches.
- However, due to the long duration of COVID-19 confinement, an organized training plan for 68 days could not be implemented for the players.
- Post-test measurements were taken 12 days (10 training session) after the end of COVID-19 confinement period.

### 2.2. Participants 

Performance measurements of 108 male youth soccer players from different age categories (U15, U16, U17, and U19) in the grassroots of a professional football team competing in the Turkish Super League were taken periodically before and after the COVID-19 lockdown and confinement. From among these 108 players, the data of the players who fitted the below eligibility criteria were utilized in the analysis. 

For being included in this study, players must have respected the following inclusion criteria: (i) had at least 5 years of experience in soccer training and performed 3–4 regular training sessions (approximately 90–120 min per session); (ii) participating in all performance measurements before and after the prolonged absence from organized training; (iii) individual exercise or personalized training programs not being performed at home during the COVID-19 confinement period; and (vii) having no previous musculoskeletal injury. 

In line with this, a total of sixty-eight male U15 (height 162.5 ± 0.07 cm; body mass 53.3 ± 8.1 kg), U16 (height 172.5 ± 0.04 cm; body mass 59.1 ± 4.8 kg), U17 (height 173.9 ± 0.08 cm; body mass 62.9 ± 6.5 kg), and U19 (height 175.3 ± 0.05 cm; body mass 66.8 ± 5.6 kg) youth soccer players were included in the study. The research was fully approved by the Ethics Committee of the local university and conformed to the recommendations of the Declaration of Helsinki. Prior to giving their written consent to participate, adult players and parents/legal guardians of youth players were fully informed about the aims, experimental procedures, and potential risks of the research. 

### 2.3. Procedures

**Figure 2:**
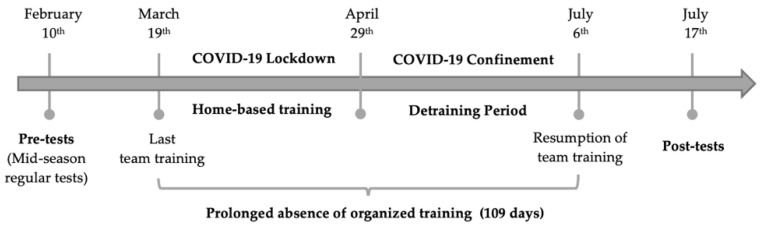
The timeline involving important dates of the study.

Pre-quarantine performance measurements of the players were taken in the mid-season (6 weeks before the lockdown began). Post-quarantine performance measurements were taken again after 15 weeks (March 2020–July 2020) in the second week of their training. The timeline involving important dates of the study is given in Figure 2.

The testing assessments were applied on two different days with 24 h of recovery in between, and 3 min of rest before the first assessment. The first day of assessment included the body composition analysis (height, weight, and body fat measurement) and the neuromuscular performance tests (vertical jump, sprint, and COD). The assessments of the first day occurred at 3:00 p.m., in a room conditioned to 23 degrees Celsius and 56% relative humidity. The observers were the same and the conditions of repeatability were ensured since the same place, conditions, and observers were ensured. A ten-minute period was provided between assessments performed at body composition and neuromuscular tests. At the second day of the assessments, an aerobic capacity test was applied under the following conditions: 04:00 p.m., 23 degrees Celsius, and 38% relative humidity. Neuromuscular performance and aerobic capacity tests were performed on artificial turf after a standardized warm-up protocol based on the RAMP method [28,29].

#### 2.3.1. Body Composition Assessment

Body mass measurements were taken pre- and post-lockdown period and the body fat percentage (BF%) was evaluated with 4-site skinfold measurement (biceps, triceps, iliac crest, and subscapular) according to the Durnin–Womersley formula [30]. At least two measurements were taken from each athlete and if there was more than 5 percent difference between the two measurements, a third measurement was taken. The assessments occurred at 3.00 p.m. of the day, at least 4 h after the last meal. 

#### 2.3.2. Neuromuscular Performances 

Vertical jump performance: Participants’ vertical jumping was evaluated with the Countermovement Jump (CMJ) with no arm sway using the Optojump optical measurement system (OptojumpNext, Microgate, Bolzano, Italy). The devices revealed a level of accuracy and precision of 92% to 98% for measuring the vertical height jump [31,32]. The participants performed three vertical trials with a 2 min recovery and the best trial was used for the analyses. During the trial, the participants were asked to jump keeping their hands on the hips and without bending the legs from take-off and landing phase. The flying time was monitored, and the height of jump was collected based on the final information provided by the device. The best height of the jumps in the three trials was collected for further statistical analysis.

Sprint performance: The 30 m sprint time was measured using an electronic timing gates system (Smartspeed, Fusion Sport, Queensland, Australia). The timing gates were positioned at 1.2 m above the floor. Players were positioned 0.5 m from the first timing gate and performed 2 trials spaced 3 min apart. The best sprinting time (lower value) was collected and used for further statistical analysis. 

**Figure 3:**
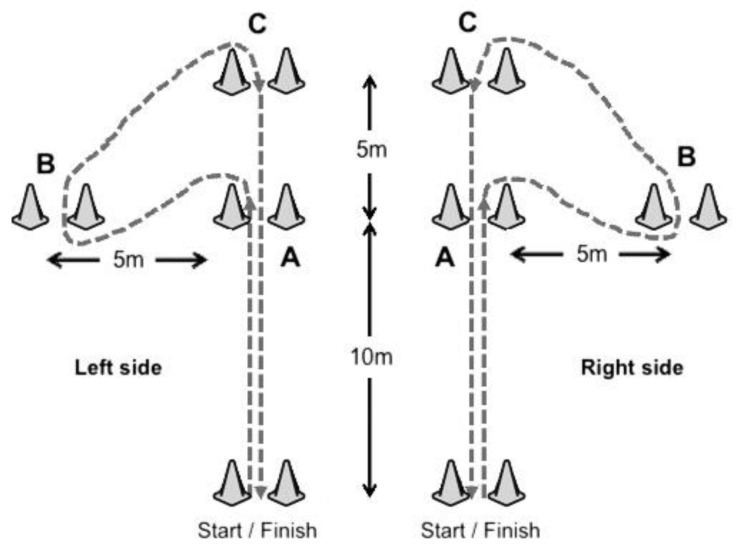
Arrowhead agility test.

Change-of-direction performance: The arrowhead agility test was measured using an electronic timing gates system (Smartspeed, Fusion Sport, Queensland, Australia) positioned at the start line 1.2 m above the floor, as described in the literature [33,34]. The participant was positioned 0.50 m from the timing gate and sprinted from the start line to the middle marker (A), turned to the left or right side to sprint around the marker (B), sprinted around the top marker (C), and sprinted back through the timing gate to finish the test (Figure 3). The test was performed for left and right sides with four randomized trials separated by at least 3 min of rest. The best trials (the smallest time to complete the task) of each side was recorded for analysis [35].

#### 2.3.3. Aerobic Capacity

The yo-yo intermittent recovery test level-1 (YYIRTL-1) was used to evaluate the aerobic fitness level of the participants. YYIRTL-1 consists of 20 m shuttle runs performed at increased velocities with 10 s of active recovery between runs. The test consisted of 4 running bouts at 10–13 km·h^−1^ (0–160 m) and another 7 runs at 13.5–14 km·h^−1^ (160–440 m), and it continues with 0.5 km·h^−1^ speed increments after every 8 running bouts (760, 1080, 1400, 1720 m, etc.) until exhaustion (Table 1). When the participants failed twice to reach the line in time, the total distance covered was considered the testing score [36,37].

#### 2.3.4. Statistical Analysis

The Shapiro–Wilk test was performed to assess normality and homogeneity for each variable. After it was determined that the data were normally distributed and homogenous, the mixed ANOVA test was performed. A mixed ANOVA (factor∗time) was conducted to investigate within-group and intra-group analysis of the data. Interaction between values was reported as *p*-value and partial eta squared. Differences between groups (between-group differences pre-post) were compared with one-way ANOVA. The effect size of the differences between the tests at two different times was determined as Cohen’s d where 0–0.19 was considered trivial, 0.20–0.59 small, 0.60–1.19 moderate, 1.20–1.99 large, 2.00–3.99 very large, and d > 4.00 extreme [38]. A significance level of *p* < 0.05 was implemented. Statistical analysis was performed using the IBM SPSS statistics software (v.25, IBM, New York, NY, USA). 

## 3. Results

**Figure 4:**
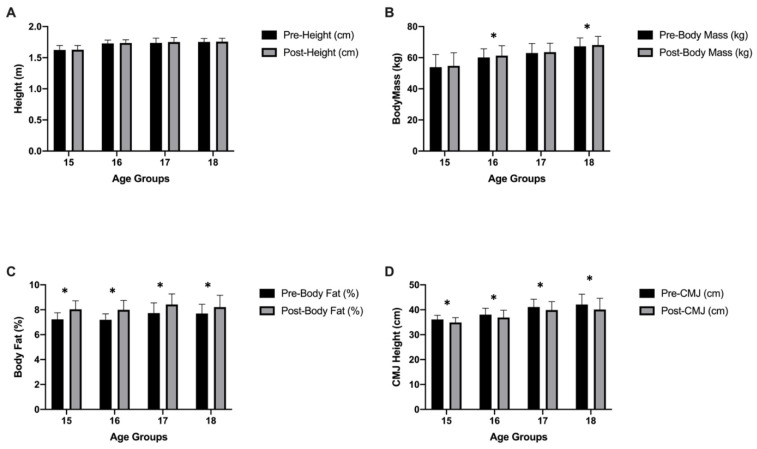
Height (**A**)**,** Body mass (**B**), Body fat percentage (**C**), and Countermovement jump (**D**) values before and after prolonged absence of organized training; * significant difference (*p* < 0.05).

**Figure 5:**
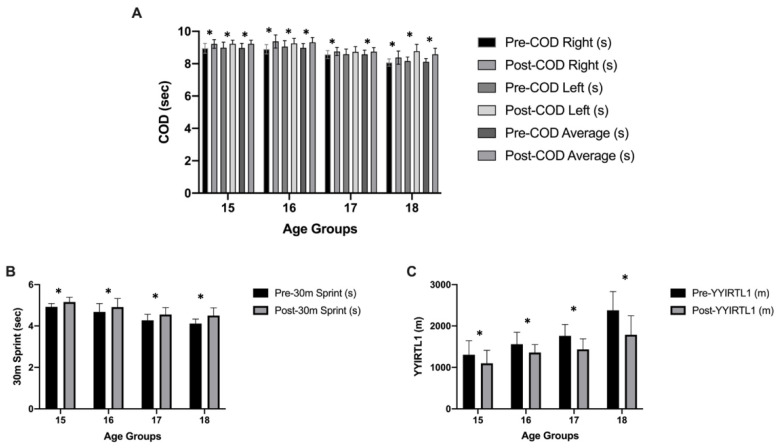
COD right, COD left and COD average (**A**), 30 m sprint (**B**), and YYIRTL-1 (**C**) values before and after prolonged absence of organized training; * significant difference (*p* < 0.05).

Descriptive statistics of body composition, neuromuscular performances and aerobic capacity values, in-group and between-group analysis values are presented in Table 2. Significant interactions were found between groups and time (pre-post) in the mixed ANOVA conducted for height (*p* = 0.001; $\eta_{p}^{2}$ ≤ 0.424), body mass (*p* = 0.001; $\eta_{p}^{2}$ ≤ 0.388), CMJ (*p* = 0.001; $\eta_{p}^{2}$ ≤ 0.354), COD right (*p* = 0.001; $\eta_{p}^{2}$ ≤ 0.622), COD left (*p* = 0.001; $\eta_{p}^{2}$ ≤ 0.472), COD average (*p* = 0.001; $\eta_{p}^{2}$ ≤ 0.615), 30 m sprint (*p* = 0.001; $\eta_{p}^{2}$ ≤ 0.492), and YYIRTL-1 (*p* = 0.001; $\eta_{p}^{2}$ ≤ 0.508). No significant interactions were found between groups and time (pre-post) in the mixed ANOVA conducted on body fat (*p* = 0.022; $\eta_{p}^{2}$ ≤ 0.071). Pre- and post-test values of height, body mass, BF%, and CMJ are indicated in Figure 4. Pre- and post-test values of COD right, COD left, COD average, 30 m sprint (s), and YYIRTL-1 (m) are indicated in Figure 5.

## 4. Discussion

Detraining is very important for understanding the many changes that negatively affect the future performance of athletes with the cessation of training [20]. Although it is stated in studies that home exercises can prevent physical performance decreases in athletes during short-term detraining such as the off-season and transition period [39,40,41], it is thought that the effects of the long-term detraining may be much more detrimental due to the COVID-19 lockdown and confinement. 

Although sport scientists, physiotherapists, and S&C coaches have many doubts about how long-term training affects soccer players’ physical performance and injury risk, all agreed that their physical performance will have declined when they return to training [42].

For this reason, to better understand the real effect of long term detraining caused by stay at home orders, the purpose of this study is to examine how body composition, neuromuscular performances, and aerobic capacity have changed after 15 weeks’ prolonged absence from organized training in youth soccer players. 

### 4.1. Body Composition

Our results indicate that body mass and body fat percentages increased significantly in all age groups due to the prolonged absence of organized training caused by stay at home orders. 

According to the literature, it has been stated that there is a significant increase in body mass in well-trained soccer players, even when they take a short break (2–3 weeks) from training [12]. 

It was determined that professional soccer players have an increase of 2% in body mass and approximately 12% in fat percentage due to the detraining during the COVID-19 lockdown compared to the traditional off-season [9]. In a study (32), conducted on 58 professional soccer players, it was determined that during the 4-week transition period without physical activity, body mass and body fat percentages increased significantly compared to the training group. Suarez-Arrones et al. [13] found that body fat increased significantly after 5 weeks of detraining [13]. It was determined that the body fat percentage of professional football players increased even though they did home-based training during the 7-week lockdown period [43]. According to Korkmaz et al. [18], there are significant changes that should be taken into account in the body fat percentage of professional soccer players after 89 days of lockdown.

Additionally, an increasing body fat percentage potentially poses a higher risk factor for muscle strain in soccer [15] and maintaining a proper body composition is crucial for neuromuscular performances [44].

### 4.2. Neuromuscular Performances

According to our findings, the CMJ, COD ability, and 30 m sprint performances of all age groups were adversely affected by a prolonged absence from organized training due to the stay at home orders.

Although it is stated that a detraining period lasting less than 4 weeks does not have significant effects on neuromuscular performance (muscle strength and power) in athletes with high fitness levels [45], it does not seem possible to say that it is the same in a long-term detraining period such as COVID-19 lockdown. 

It was stated that the highest performance loss after the 8-week lockdown period was in the 20-m sprint and COD test results in young basketball players [16].

In a study [9], comparing the traditional off-season and the 63-day quarantine period, it was determined that there were significant detrimental effects in the CMJ and 10–20 m sprint performances in professional soccer players. 

Considering these findings, neuromuscular changes such as CMJ, COD ability and sprint performances should be carefully examined in order to ensure optimum performance development and prevent injuries to athletes when returning to soccer training after long-term detraining [5,8,9,17]. 

### 4.3. Aerobic Capacity

According to our findings, the highest rate of performance loss caused by long-term detraining due to COVID-19 stay at home orders is in the aerobic capacity of youth soccer players for all age groups.

With respect to previous studies [6,9,12,46], significant decreases were found in the maximal oxygen consumption values of soccer players in the off-season period. It has been determined that this rate is approximately 6% in the 4-week transition period in soccer players [39]. 

It is observed that despite performing 30 min of aerobic exercise 3 days a week during the 40 days of the lockdown period, professional soccer players’ running distances (YYIRTL-1) and aerobic capacity had decreased [47]. 

In another study conducted on handball players during the COVID-19 lockdown, although a structured 9-week home-based exercise program preserved the neuromuscular performance of the athletes, it was insufficient to maintain their aerobic capacity [48]. 

It is seen that there are similar results in many studies where aerobic capacity is evaluated before and after the COVID-19 lockdown [49,50]. 

When pre- and post-60-day lockdown performance changes of La Liga soccer players during matches was measured, the most significant loss was observed in total distance running [51]. 

### 4.4. Study Limitations and Future Research

This study has some limitations. One of the limitations is the absence of a control group. Considering that most of the players followed similar behaviors across the time of exposure, it was not possible to differentiate them into two groups, nor to associate the exposures to the outcomes. However, it was possible to create differences between age groups. Another limitation was that the athletes were not monitored for dietary intake and physical activity levels during the stay-at-home orders. Although the indications for being at home, it is possible that some physical activity variations may occur based on the opportunities which home and region have. Moreover, it is still unknown how the specific baseline levels or genetics can influence the final changes in physical fitness. Thus, future research about detraining or training cessation should inspect the effects moderators and mediators such as genetics, baseline levels, trainability, physical activity patterns, or dietary intake can have on the final changes in physical fitness. Moreover, future studies should consider analyzing the effects of minimum effective dose in youth soccer players, which could offer an alternative to easily implement the training process in constrained contexts such as at home. 

#### Practical Implications

Considering the evidence of a significant decrement of body composition, aerobic capacity, and neuromuscular performance, it is important to advance an individualized training process that may include easy-to-use and effective methods such as high-intensity interval training, strength training based on bodyweight, and reactive strength as plyometrics that can be used in any place without specific instruments and can offer a way to mitigate the effects of long-term training cessation.

## 5. Conclusions

To our knowledge, this is the first study carried out based on the real scenario caused by stay at home orders with a large number of participants from different age groups in young soccer players. 

As a conclusion, it is obvious that there is a detrimental effect on body composition, neuromuscular performances, and particularly aerobic capacity following a prolonged period (15 weeks) of detraining because of stay at home orders. Compared to short- and long-term detraining, it is important to consider that the neuromuscular performances and aerobic capacities of young soccer players are more affected by stay at home orders. 

The difficulty in controlling the physical activity status of a large number of young football players from different age groups may be a limitation of this research and it is important to consider this when interpreting the research results. Although soccer players were asked whether they participated in regular exercise, there is always a possibility that some players might have given biased answer for different reasons. Another limitation of the present study is that we do not compare the body composition and performance measurements of youth soccer players immediately before and after the COVID-19 stay at home orders. 

Additionally, it is important to evaluate neuromuscular performances such as flexibility, muscular strength, and repeated sprint in terms of training planning and especially preventing injuries after a long period of detraining in youth soccer players. 

On the other hand, the performance divergences among soccer players of different ages and competitive levels might be an outcome of differences in the performance levels they had during the pre-pandemic period, the length of lockdown period they experienced, and the individual- and home-based exercise they carried out during the lockdown.

## Figures and Tables

**Table 1:** Yo-yo intermittent recovery level-1 test protocol.

Stage	Speed (km·h^−1^)	Shuttle Bouts (2 × 20 m)	Split Distance (m)	Accumulated Distance (m)
1	10	1	40	40
2	12	1	40	80
3	13	2	80	160
4	13.5	3	120	280
5	14	4	160	440
6	14.5	8	320	760
7	15	8	320	1080
8	15.5	8	320	1400
9	16	8	320	1720
10	16.5	8	320	2040
11	17	8	320	2360
12	17.5	8	320	2680
13	18	8	320	3000
14	18.5	8	320	3320
15	19	8	320	3640

**Table 2:** Descriptive statistics (mean and standard deviation), within- and between-group analysis.

Variables	15 Age (within Group Analysis)				16 Age (within Group Analysis)				17 Age (within Group Analysis)				18 Age (within Group Analysis)				Between-Group Differences (Pre)		Between-Group Differences (Post)	
	Pre	Post	*p*	d	Pre	Post	*p*	d	Pre	Post	*p*	d	Pre	Post	*p*	d	*p*	$\eta_{p}^{2}$	*p*	$\eta_{p}^{2}$
Height (cm)	1.62 ± 0.07	1.62 ± 0.06	0.331	−0.236	1.72 ± 0.05	1.73 ± 0.05	0.009	−0.716	1.73 ± 0.07	1.75 ± 0.07	0.086	−0.496	1.75 ± 0.05	1.75 ± 0.05	0.119	−0.365	≤0.001 *	0.407	0.001	0.442
Body Mass (kg)	53.92 ± 8.12	54.79 ± 8.38	0.017	−0.625	60.18 ± 5.54	61.29 ± 6.37	0.003 *	−0.830	62.97 ± 6.14	63.58 ± 5.72	0.080	−0.507	67.31 ± 5.37	68.13 ± 5.47	0.005 *	−0.710	≤0.001 *	0.399	≤0.001	0.378
Body Fat (%)	7.23 ± 0.52	8.03 ± 0.68	≤0.001 *	−2.038	7.19 ± 0.47	7.99 ± 0.75	≤0.001 *	−1.812	7.72 ± 0.82	8.42 ± 0.84	≤0.001 *	−1.603	7.69 ± 0.74	8.21 ± 0.94	0.002 *	−0.809	0.025	0.133	0.459	0.039
CMJ (cm)	36.08 ± 1.70	34.87 ± 1.96	0.003 *	0.824	38.07 ± 2.50	36.88 ± 2.94	≤0.001 *	1.020	41.10 ± 3.12	39.88 ± 3.36	0.009 *	0.827	42.09 ± 4.19	40.08 ± 4.53	≤0.001 *	2.048	≤0.001 *	0.403	≤0.001 *	0.310
COD Right (s)	8.95 ± 0.30	9.22 ± 0.26	0.005 *	−0.756	8.89 ± 0.28	9.37 ± 0.40	≤0.001 *	−1.211	8.56 ± 0.24	8.75 ± 0.24	≤0.001 *	−1.192	8.07 ± 0.22	8.37 ± 0.40	≤0.001 *	−0.904	≤0.001 *	0.664	≤0.001 *	0.599
COD Left (s)	8.98 ± 0.34	9.22 ± 0.22	≤0.001 *	−1.074	9.05 ± 0.36	9.25 ± 0.30	0.012 *	−0.687	8.58 ± 0.31	8.73 ± 0.32	0.067	−0.533	8.16 ± 0.25	8.77 ± 0.42	≤0.001 *	−1.253	≤0.001 *	0.588	≤0.001 *	0.362
COD Average (s)	8.97 ± 0.28	9.22 ± 0.23	≤0.001 *	−1.061	8.97 ± 0.27	9.31 ± 0.30	≤0.001 *	−1.501	8.58 ± 0.25	8.74 ± 0.25	0.006 *	−0.885	8.12 ± 0.20	8.57 ± 0.37	≤0.001 *	−1.287	≤0.001 *	0.694	≤0.001 *	0.543
30 m Sprint (s)	4.91 ± 0.16	5.15 ± 0.22	≤0.001 *	−1.600	4.67 ± 0.40	4.91 ± 0.41	≤0.001 *	−1.055	4.27 ± 0.29	4.55 ± 0.33	≤0.001 *	−1.188	4.11 ± 0.21	4.50 ± 0.37	≤0.001 *	−1.294	≤0.001 *	0.592	≤0.001 *	0.400
YYIRTL-1 (m)	1305 ± 339	1098 ± 315	≤0.001 *	0.949	1562 ± 286	1358 ± 192	≤0.001 *	1.130	1760 ± 275	1437 ± 251	0.002 *	1.047	2379 ± 452	1789 ± 459	≤0.001 *	1.169	≤0.001 *	0.597	≤0.001 *	0.395

CMJ: Countermovement jump test; COD: change-of-direction *: *p*-value < 0.05.

## Data Availability

The datasets used and/or analyzed during the current study are available from the corresponding author on reasonable request.
